# Adipocyte-Derived Small Extracellular Vesicles from Patients with Alzheimer Disease Carry miRNAs Predicted to Target the CREB Signaling Pathway in Neurons

**DOI:** 10.3390/ijms241814024

**Published:** 2023-09-13

**Authors:** Rachael A. Batabyal, Ankush Bansal, Laura Reck Cechinel, Kayla Authelet, Madeleine Goldberg, Evan Nadler, C. Dirk Keene, Suman Jayadev, Kimiko Domoto-Reilly, Gail Li, Elaine Peskind, Kazue Hashimoto-Torii, Dedra Buchwald, Robert J. Freishtat

**Affiliations:** 1 Center for Genetic Medicine, Children’s National Hospital, Washington, DC 20012, USAmsg7968@nyu.edu (M.G.); rfreishtat@uncommoncures.com (R.J.F.); 2 School of Medicine and Health Sciences, The George Washington University, Washington, DC 20037, USA; evan.nadler@verizon.net (E.N.);; 3 Center for Neuroscience Research, Children’s National Hospital, Washington, DC 20010, USA; 4 Division of Pediatric Surgery, Children’s National Hospital, Washington, DC 20010, USA; 5 Department of Pathology, University of Washington, Seattle, WA 98104, USA; cdkeene@uw.edu; 6 Department of Neurology, University of Washington, Seattle, WA 98104, USA; sumie@uw.edu (S.J.);; 7 Department of Psychology and Behavioral Sciences, School of Medicine, University of Washington, Seattle, WA 98104, USA; 8 Veterans Affairs Puget Sound Health Care System, Seattle, WA 98108, USA; 9 Institute for Research Education to Advance Community Health, Elson S. Floyd College of Medicine, Washington State University, Spokane, WA 99202, USA

**Keywords:** exosomes, dementia, adipose tissue, EVs, obesity

## Abstract

Alzheimer disease (AD) is characterized by amyloid-β (Aβ) plaques, neurofibrillary tangles, synaptic dysfunction, and progressive dementia. Midlife obesity increases the risk of developing AD. Adipocyte-derived small extracellular vesicles (ad-sEVs) have been implicated as a mechanism in several obesity-related diseases. We hypothesized that ad-sEVs from patients with AD would contain miRNAs predicted to downregulate pathways involved in synaptic plasticity and memory formation. We isolated ad-sEVs from the serum and cerebrospinal fluid (CSF) of patients with AD and controls and compared miRNA expression profiles. We performed weighted gene co-expression network analysis (WGCNA) on differentially expressed miRNAs to identify highly interconnected clusters correlating with clinical traits. The WGCNA identified a module of differentially expressed miRNAs, in both the serum and CSF, that was inversely correlated with the Mini-Mental State Examination scores. Within this module, miRNAs that downregulate CREB signaling in neurons were highly represented. These results demonstrate that miRNAs carried by ad-sEVs in patients with AD may downregulate CREB signaling and provide a potential mechanistic link between midlife obesity and increased risk of AD.

## 1. Introduction

The number of people aged 65 and older with Alzheimer disease (AD) in the United States is 6.5 million and is expected to reach 13.8 million by 2060. Alzheimer disease is the 7th leading cause of death in the United States [1]. It is a neurodegenerative disease characterized pathologically by amyloid beta (β) plaques and neurofibrillary tangles in the brain and clinically by the insidious onset of progressive dementia [2]. Amyloid beta (Aβ) and neurofibrillary tangles are the neuropathologic changes classically associated with AD; however, given the importance of synaptic plasticity and maintenance in learning and memory, synaptic dysfunction has been studied as a mechanism of dementia in AD and correlates with clinical dementia more closely than amyloid beta plaques or neurofibrillary tangles [3,4,5,6,7,8]. 

Several modifiable risk factors for dementia share obesity as a causal factor, including cardiovascular disease and type 2 diabetes mellitus [9,10]. Obesity, like AD, is associated with decreased synaptic plasticity and is a known risk factor for AD [11,12,13]. In addition, midlife obesity has been associated with up to a threefold increased risk of developing AD [13,14,15,16]. This suggests an adipose-derived mediator of disease; however, the biological mechanisms underlying this risk factor are largely unknown. 

Adipose tissue, once thought of as a mere storage depot for energy, is the largest endocrine organ in the body and a key regulator of energy homeostasis and inflammation [17,18]. A more recently discovered means of interorgan signaling by adipose tissue is via adipocyte-derived small extracellular vesicles (ad-sEVs) [19,20,21]. sEVs, sometimes called exosomes, are a heterogeneous group of cellular-derived, membrane-bound particles < 150 nm in size that are released by almost all cell types [22]. Adipocyte-derived small extracellular vesicles (ad-sEVs) are released from adipocytes into the serum, where they carry cargo to other organs [23]. These nanoparticles contain various cargo, including lipids, proteins, sugars, and nucleic acids. Ad-sEVs constitute a significant source of circulating sEV microRNA (miRNA) [23,24]. Ad-sEVs have been implicated in the development of peripheral insulin resistance, metabolic-associated fatty liver disease, and dyslipidemia [19,20,21,25]. However, the role of ad-sEVs in the development of neurodegenerative diseases such as AD has not been well studied. 

Given the role that ad-sEVs play in many obesity-related diseases, the increased risk of AD among patients with midlife obesity, and the known alterations in synaptic plasticity in both obesity and AD, we hypothesized that ad-sEVs from patients with AD would contain miRNAs predicted to downregulate pathways involved in synaptic plasticity and memory formation. To test this hypothesis, we isolated ad-sEVs from the serum and CSF of patients (60–89 years old) with AD and controls obtained from the University of Washington Alzheimer Disease Research Center clinical core longitudinal study and collaborating research centers. We compared the miRNA expression profiles of those with AD and controls. We performed weighted gene co-expression network analysis (WGCNA) on differentially expressed miRNAs to identify highly interconnected clusters correlating with clinical traits and pathway analysis to perform in silico prediction of their biological functions.

## 2. Results

### 2.1. Subject Demographics

Ad-sEV miRNA was isolated from the serum and cerebrospinal fluid (CSF) of 18 patients with probable AD and 14 control patients. Clinical characteristics between groups did not differ (*p* > 0.05) except for Clinical Dementia Rating and CSF Aβ_1–42_. Additional selected clinical features are shown in Table 1.

### 2.2. miRNA Profiling from ad-sEVs from Serum and CSF Identified Differences between Disease States

**Figure 1:**
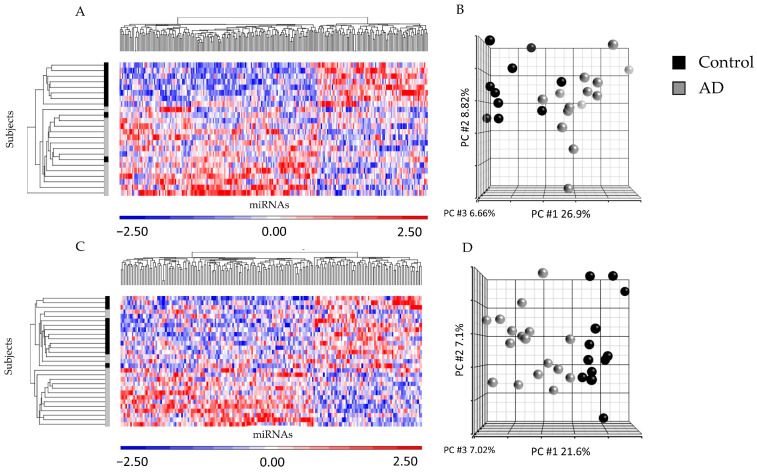
Hierarchical clustering and principal component analysis plots comparing ad-sEV microRNA from patients with AD and non-AD controls. (**A**). The heatmap of the differentially expressed microRNAs identified in the ad-sEVs isolated from the CSF demonstrates almost complete separation of patients with AD (gray) and controls (black) along the y-axis on the left of the heatmap. The clustering of the differentially expressed miRNAs can be found along the top of this heatmap. The color bar represents the standardized expression of each microRNA to a mean of 0. Upregulated microRNAs have positive values and are displayed in red. Downregulated microRNAs have negative values and are displayed in blue. (**B**). The PCA plot from the CSF demonstrates that subjects cluster according to their disease state (AD vs. control). Each dot represents the overall microRNA expression of a single subject. Black represents control subjects, and gray represents subjects with AD. (**C**). The heatmap of the differentially expressed microRNAs identified in the ad-sEVs isolated from the serum demonstrates almost complete separation of patients with AD (gray) and controls (black) along the y-axis on the left of this heatmap. The clustering of the differentially expressed miRNAs can be found along the top of the heatmap. (**D**). The PCA plot from the serum demonstrates that subjects cluster together according to their disease state.

There was a strong linear correlation between miRNA isolated from ad-sEVs from the serum and the CSF (Supplemental Figure S1). When serum and CSF were analyzed separately, between the AD and controls, there were 251 differentially expressed miRNA in the CSF (Supplemental Table S1) and 189 differentially expressed miRNA in the serum (FC ≥ |1.1|, *p* < 0.1) (Supplemental Table S2). In the unsupervised hierarchical clustering of the differentially expressed miRNA in the CSF (Figure 1A) and serum (Figure 1C), the miRNA expression profiles showed almost complete separation of patients with AD and controls. Similarly, in the principal component analysis (PCA) plot, the expression profiles almost completely separated patients with AD from controls (Figure 1B,D). 

### 2.3. Differentially Expressed miRNA in Serum and CSF Are Predicted to Downregulate the CREB Signaling Pathway in Neurons

The predicted cumulative effects of the differentially expressed miRNAs from ad-sEVs from the serum and CSF on gene expression were analyzed. Using QIAGEN Ingenuity Pathway Analysis (IPA) [26], we performed a core analysis to examine the predicted effects of the differentially expressed ad-sEV miRNA in the serum (AD vs. controls) and the CSF (AD vs. controls). For this analysis, serum and CSF were analyzed independently. Of the 7765 mRNAs targeted by the ad-sEV miRNAs isolated from the CSF, 6538 were predicted to be downregulated, and 1227 were predicted to be upregulated. Of 4718 mRNA targeted by the ad-sEV miRNAs in the serum, 3954 were predicted to be downregulated, and 764 were predicted to be upregulated. 

**Figure 2:**
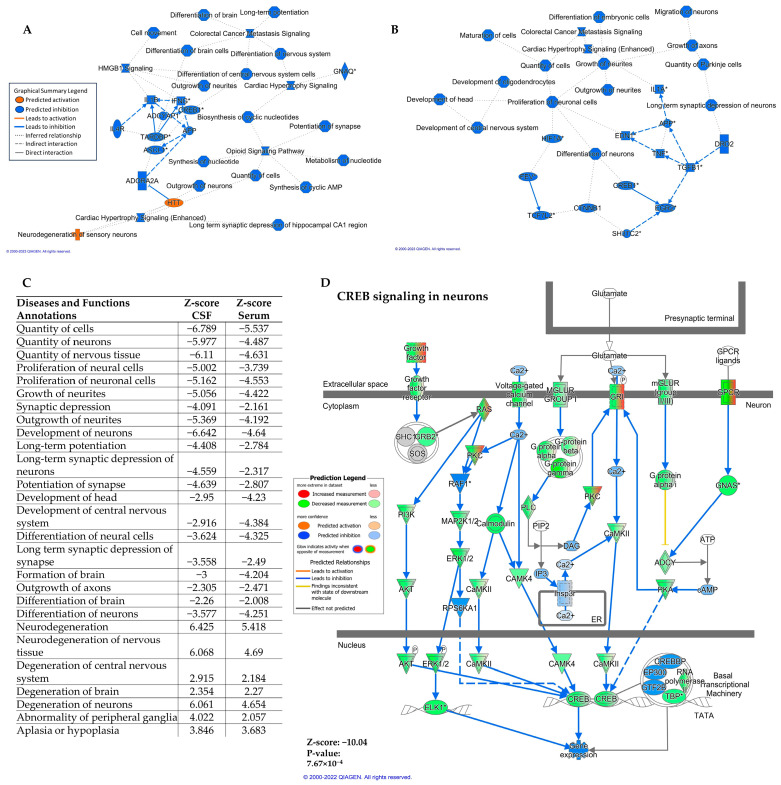
Pathway analysis of differentially expressed ad-sEV miRNA (AD vs. control). (**A**). Graphical summary of IPA analysis illustrating the predicted effects of differentially expressed cerebrospinal fluid (CSF) ad-sEV miRNAs. The analysis indicates downregulation of long-term potentiation and long-term synaptic depression, showing potential modulation of synaptic plasticity. Additionally, the downregulation of Cyclic AMP response element binding protein (CREB) 1 was observed. (**B**). Graphical summary of the predicted effects of differentially expressed serum ad-sEV miRNAs. This analysis showed downregulation of central nervous system development, cell maturation, neurite outgrowth, and long-term synaptic depression in neurons. (**C**). Diseases and functions annotations predicted by differentially expressed miRNA isolated from ad-sEVs in the CSF and serum. A positive z-score indicates predicted activation, and a negative z-score indicates predicted downregulation. (**D**). Pathway analysis revealed that the CREB signaling pathway was predicted to be downregulated by the microRNA in ad-sEVs isolated from CSF. In CSF ad-sEVs, 343 out of 584 molecules in CREB signaling were targeted by the differentially expressed miRNAs in AD patients vs. controls. * Denotes that multiple identifiers in the dataset map to a single gene in the global molecular network.

The graphical summary (Figure 2A) of the predicted effects of these differentially expressed CSF ad-sEV miRNAs demonstrated several processes of interest, including downregulation of long-term potentiation and long-term synaptic depression, indicating that these miRNAs were predicted to affect synaptic plasticity, which is the ability of the synapses to modify their strength and structure and the process that underlies memory formation. Cyclic AMP response element binding protein (CREB) 1 was also predicted to be downregulated. The graphical summary of the serum analysis demonstrated similar results with predicted downregulation of CNS development, maturation of cells, outgrowth of neurites, and long-term synaptic depression of neurons (Figure 2B). Like in CSF, CREB1 was also predicted to be downregulated by the serum ad-sEV miRNAs. We then examined several diseases and functions predicted to be affected by the miRNAs contained by the ad-sEVs. Figure 2C demonstrates all predicted processes common to both the serum and CSF analyses. Neural cell proliferation, differentiation, and development were predicted to be downregulated. Critical to AD pathophysiology, both long-term potentiation and long-term depression [27] were predicted to be downregulated. Of note, in the CSF, long-term depression in the hippocampus, specifically, was predicted to be downregulated. In both serum and CSF, neurodegeneration was predicted to be activated.

Since decreased CREB signaling in neurons is well described in AD, induces synaptic dysfunction, and has been implicated in the development of memory impairment [8], we examined the predicted effects of the differentially expressed miRNAs on this pathway (Figure 2D). Downregulation of CREB signaling was predicted by the ad-sEVs isolated both from the CSF (Figure 2D) and the serum (Supplementary Figure S2). In the CSF, 343 of 584 molecules were predicted to be downregulated (z-score = −10, *p* = 0.0007). In the serum, 338 of 584 molecules were predicted to be downregulated (z-score = −8.7, *p* = 0.02). 

### 2.4. Identification and Functional Analysis of miRNA Clusters That Inversely Correlate with MMSE Scores

**Figure 3:**
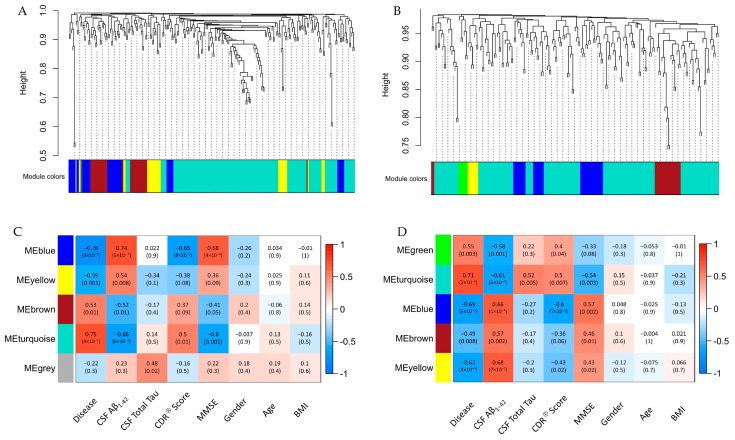
WGCNA analysis. miRNA dendrogram showing the co-expression modules in the differentially expressed miRNA isolated from ad-sEVs in the (**A**) CSF and (**B**) serum (AD vs. control FC ≥ |1.1|, *p* < 0.1). Highly correlated miRNAs are grouped into colored modules. Table of correlation between clinical traits and miRNA modules from the (**C**) CSF and (**D**) serum. Each row represents a miRNA module, and each column represents a clinical trait.

To identify highly interconnected miRNA clusters among these differentially expressed miRNAs and correlate the phenotypic data with these clusters, we performed a weighted gene co-expression network analysis (WGCNA). Hierarchical clustering identified five distinct co-expression modules in the CSF (blue, yellow, brown, turquoise, and gray) and five in the serum (green, turquoise, blue, brown, and yellow) (Figure 3A,B). In addition, correlation with relevant phenotypic traits was performed (Figure 3C,D). Clinically, we were most interested in the correlation between miRNA modules and cognitive testing scores, and we specifically looked for a negative correlation between miRNA module and Mini-Mental State Examination (MMSE) score. The turquoise module showed the most negative correlation in the CSF (−0.6, *p* = 0.002) and in the serum (−0.54, *p* = 0.003). 

To explore the potential functional role of the miRNAs within each turquoise module, we identified hub miRNAs for each module and performed pathway analysis on these miRNAs based on the differential expression in samples from AD and controls (FC ≥ |1.1|, *p* < 0.1). In the CSF, the turquoise module contained 76 hub miRNAs (module membership score ≥ 0.5). The serum turquoise module contained 27 hub miRNAs. The concordant pathways that were predicted to be most affected based on the z-score (Z score ≥ |2|) in the serum and CSF are listed in Table 2. Among these pathways, CREB signaling in neurons was predicted to be most downregulated in both serum and CSF. The CREB1 signaling in neurons pathway was a target for 36 out of 76 (47%) hub miRNAs in the CSF turquoise module and 24 out of 27 (89%) hub miRNAs in the serum turquoise module. There were two differentially expressed miRNAs, miR-6760-3p (FC 1.6, *p*-value < 0.05 in CSF ad-sEVs, FC 1.5, *p*-value < 0.05 in serum ad-sEVs) and miR-6798-3p (FC 1.8, *p*-value < 0.05 in CSF ad-sEVs, FC 1.4, *p*-value < 0.05 in serum ad-sEVs), which were found in the turquoise module in both the serum and CSF and are predicted to target the CREB signaling pathway. miR-6760-3p is predicted to target *GPR26* and *HTR1F*. miR-6798-3p is predicted to target *ADRA1D*, *GPR153*, and *HTR1E*.

## 3. Discussion

This study investigated the miRNA expression profiles of ad-sEVs in the serum and CSF of patients with AD and compared them to those from control patients. We identified differentially expressed miRNAs in the ad-sEVs and utilized WGCNA and IPA to predict their biological functions. Our work highlights a potential role for ad-sEVs in the development of altered synaptic plasticity and pathways involved in memory formation in patients with AD. These data suggest that patients with dysfunctional adipose tissue, such as those with midlife obesity, have an increased risk of developing cognitive impairments. Although previous reports have identified EVs as potential carriers of important molecules involved in AD pathophysiology, including Aβ and tau [28,29,30], and several differentially expressed miRNAs have been identified in the blood, brain, and CSF of patients with AD, this work identifies that ad-sEVs can be isolated from both serum and CSF of patients with AD and are a novel mechanism of delivering potentially pathogenic miRNAs from adipocytes to the CSF. 

Increasing evidence points to a potential role of ad-sEVs in several other obesity-related diseases. For example, ad-sEVs have been implicated in the development of non-alcoholic fatty liver disease, insulin resistance, and atherosclerosis [31,32,33]. However, little research has been conducted to investigate the role of ad-sEVs in the development of neurocognitive decline, as occurs in AD. 

Our study demonstrated that ad-sEVs from patients with AD carry miRNAs that are predicted to target the CREB signaling pathway in neurons. Furthermore, WGCNA results indicated that miRNAs that target the CREB pathway are highly represented within the miRNA modules that negatively correlate with MMSE scores. CREB is a nuclear transcription factor for molecules involved in neuronal survival, maintenance of long-term potentiation, synaptic plasticity, learning, and memory processes [34]. Restoring its function can reverse the learning and memory deficits in mouse models of AD [35]. Evidence from post-mortem brain tissue demonstrates decreased total and phosphorylated CREB in AD [36,37], and Pugazhenthi et al. [38] demonstrated that, in AD, a chronic downregulation of CREB-mediated transcription leads to decreased CREB in hippocampal neurons. Aβ has also been implicated in downregulating CREB signaling [39]. Our findings indicate that ad-sEVs may also contribute to this downregulation of CREB and the CREB signaling pathway in AD. In animal models, a high-fat diet also inhibits CREB signaling [40]. Taken together, this suggests that ad-sEVs are involved in downregulating CREB signaling in obesity, which may place those with obesity at a higher risk of developing cognitive impairment in AD. Previous research has shown that adipose-derived EVs from mice and humans with type 2 diabetes mellitus can mediate changes in synaptic plasticity in mice [41]. However, our study provides valuable context for clinical studies by demonstrating that ad-sEVs from patients with AD carry miRNAs that may downregulate the CREB signaling pathway.

CREB itself is a transcription factor for Brain-derived Neurotrophic Factor (BDNF). This molecule plays a central role in neuronal survival and synaptic plasticity and is decreased in patients with AD [42,43]. A study by Wang et al. [41] demonstrated that, in mice, adipose tissue-derived EVs from patients with type 2 diabetes mellitus induce cognitive impairment through the downregulation of BDNF by miR-9-3p, which in turn decreases synapse formation and synaptic plasticity and maintenance [41]. While our findings did not identify miR-9-3p as an upregulated miRNA in ad-sEVs from patients with AD, it did identify miRNAs that target the expression of G-protein coupled receptor subunits, PKA, ERK, and CREB, all members of the CREB signaling pathway that are upstream of BDNF. Furthermore, two of the miRNAs that were upregulated in the ad-sEVs from both the serum and CSF, miR-6760-3p and miR-6798-3p, have previously been described to be upregulated in the cortex of the brain in patients with AD [44]. Our findings and those by Wang et al. [41] support the idea that changes in the miRNAs released by adipocytes may contribute to cognitive impairment involved in various diseases, including diabetes and AD. Notably, several drugs specifically inhibit phosphodiesterases (i.e., rolipram and roflumilast) and increase cAMP levels, leading to a signaling cascade that increases CREB phosphorylation. However, despite demonstrated clinical efficacy, these drugs have a side effect profile that has kept them from being approved for AD [45]. Our results indicate that miRNAs carried by ad-sEVs in patients with AD may be downregulating CREB signaling, a known drug target in AD and a key pathway involved in neuronal survival, synaptic plasticity, learning, and memory. These novel findings provide a potential mechanism linking midlife obesity and the risk of AD. 

## 4. Materials and Methods

### 4.1. Study Participants and Sample Collection

Participants were recruited from the University of Washington Alzheimer Disease Research Center, the VA at Puget Sound, and collaborating AD centers. All study procedures were approved by their respective institutional review boards, and all study participants provided written informed consent. Study participants underwent extensive clinical, neuropsychological, and laboratory evaluations. History was obtained from normal subjects or from informants if patients had dementia or mild cognitive impairment. Participants were categorized as having no cognitive impairment, mild cognitive impairment, or dementia based on standard criteria [46,47]. Clinical diagnosis of AD was made at a consensus conference [48]. AD was defined as a clinical diagnosis of AD, a clinical dementia rating ≥ 0.5, and CSF Aβ_1–42_ ≤ 192 pg/mL. The specimens used as controls in the current study were obtained from the ADRC cohort at the University of Washington. ADRC cohort controls include healthy individuals recruited from community medicine clinics. Inclusion criteria for controls in the ADRC include age greater than 65, normal neuropsychological evaluations, and no personal medical history of neurodegenerative disease, brain malignancy, uncontrolled diabetes, uncontrolled hypertension, or untreated psychiatric disease. Controls in the present study were subjects without a clinical diagnosis of AD, clinical dementia rating ≤ 0.5, CSF Aβ_1–42_ > 192 pg/mL, and CSF total tau ≤ 93 pg/mL. All participants described as controls in the present study were Alzheimer’s unaffected controls, but one participant had mild cognitive impairment without a clinical diagnosis of AD. CSF was collected by lumbar puncture [47]. Blood was collected by venipuncture from all participants and centrifuged to obtain serum. All CSF and serum samples were stored at −80 °C until analysis. CSF samples were analyzed for Aβ_1–42_ and total tau using multiplexed Luminex reagents (Fujirebio, Malverne, PA, USA). 

### 4.2. Adipocyte-Derived Small Extracellular Vesicle Isolation

600 μL of serum and 500 μL of CSF per subject were thawed over ice and then centrifuged at 3000× *g* for 15 min to remove cellular debris. The supernatant was filtered through a 0.2 µm filter. Small extracellular vesicles were obtained from the serum using Exoquick Precipitation Solution (System Biosciences, Mountain View, CA, USA) and from the CSF using Exoquick TC Precipitation Solution (System Biosciences, Mountain View, CA, USA), per the manufacturer’s protocol. Ad-sEVs were selected for using fatty acid binding protein 4 (FABP4) antibody, a sensitive and specific marker for ad-sEVs, and dextran-coated magnetic particles (StemCell Technologies, Vancouver, BC, Canada). 

### 4.3. RNA Extraction and Amplification

Ad-sEV total RNA was extracted using mirVana microRNA Isolation kits (Life Technologies, Carlsbad, CA, USA). Total RNA was amplified with the Complete Seramir Exosome RNA Amplification Kit (System Biosciences, Mountainview, CA, USA). RNA quality was assessed on a subset of these samples using the Bioanalyzer 2100 (Agilent Technologies, Santa Clara, CA, USA) and Nanodrop 2000 (ThermoFisher Scientific, Waltham, MA, USA). RNA concentration was measured using a Qubit RNA Broad Range Assay Kit (Thermo Fisher Scientific, Waltham, MA, USA). 

### 4.4. Adipocyte-Derived Small EV microRNA Profiles

1000 ng of RNA was labeled with Affymetrix FlashTag Biotin HSR RNA Labeling Kit (Affymetrix, Santa Clara, CA, USA) per the manufacturer’s instructions. Labeled RNA was hybridized to GeneChip microRNA 4.0 arrays (Affymetrix, Santa Clara, CA, USA). Chips were run using a Fluidics Station 450 Protocol (Affymetrix, Santa Clara, CA, USA). The results were exported to Partek Genomics Suite (version 6.6, Partek, St. Louis, MO, USA) for analysis. For initial comparison, the entire dataset was normalized using RMA-DABG in Expression Console, and data were exported to Partek Genomics Suite. Using this data, the mean probe intensities for each of the 2578 mature miRNA probes in the CSF and serum were plotted for all paired samples (n = 21). Next, the raw data from Expression console was exported, and normalization was carried out within each biofluid using RMA-DABG. Mature human miRNAs were analyzed and compared between disease groups in Partek Genomics Suite. ANOVA was used to compare miRNAs between patients with AD and controls. Serum and CSF were analyzed separately [49]. 

### 4.5. Weighted Gene Co-Expression Network Analysis

We first identified differentially expressed ad-sEV miRNAs between patients with AD and controls using one-way ANOVA (*p* < 0.1, FC ≥ |1.1|). To explore the relationship between these differentially expressed miRNAs and the MMSE scores, we utilized weighted gene co-expression network analysis (WGCNA). This method uses eigengene network methodology to explore the relationships between external sample traits and clusters of genes. The WGCNA R package (R version 4.0.5, WGCNA version 1.72-1) was downloaded from the Comprehensive R Archive Network (CRAN) repository, and required packages were downloaded from Bioconductor to build the weighted co-expression network [50,51]. Hierarchical clustering with the Euclidean distance was used to screen for any sample outliers, and none were found in our dataset. A heatmap was constructed to visualize how clinical traits relate to the sample dendrograms within each biofluid (Supplementary Figure S3A,D). Scale independence and mean connectivity were plotted as functions of soft thresholding power (β). Maximum scale-free topology model fit was attained around a soft-thresholding power of four in the CSF and three in the serum (Supplementary Figure S3B,C,E,F). A minimum module size of three was chosen for each biofluid. Using these criteria, five miRNA modules were identified in each biofluid. We were clinically most interested in MMSE scores. We had MMSE scores available in the patients with AD and only Montreal Cognitive Assessment (MoCA) scores for controls. In order to use these cognitive test scores for this analysis, it was necessary to convert the MoCA scores to MMSE scores using a validated conversion table [52]. Next, the module eigengenes were correlated with clinical traits. We identified all miRNAs with a module membership score >0.5 as hub miRNAs. Module membership score is the correlation of the module eigengene and miRNA expression profile. For each hub miRNA, we used the differential expression data between AD and control patients (FC and *p*-value) in IPA. In brief, miRNA targets were identified using the miRNA target filter, and a core analysis was completed on these targets. Canonical pathways were then explored to understand the functional relevance of hub miRNAs in each module. Canonical pathways were considered significant if the *p*-value was >0.5 and the z-score was >2. 

### 4.6. Statistical Analysis

Principal component analysis and hierarchical clustering were performed using Partek genomics suite (version 6.6, Partek, St. Louis, MO, USA). One-way Analysis of Variance (ANOVA) was used to identify mature microRNAs that were differentially expressed between AD and control patients (*p* < 0.1, FC ≥ |1.1|) for the WGCNA analysis. Pathway Analysis and Diseases and Function annotation were conducted using QIAGEN IPA (version 94302991, QIAGEN Inc., Redwood City, CA, USA https://digitalinsights.qiagen.com/IPA) [26]. For this analysis, we uploaded all differentially expressed miRNA with a FC ≥ |1.2| and a *p*-value < 0.1 to keep to an allowable number of mRNA targets for predicted or experimentally observed mRNAs. This software uses experimentally observed miRNA–mRNA interactions from Tarbase and miRecords and predicted interactions from TargetScan. It also integrates miRNA-related findings from the Ingenuity knowledge base, which includes thousands of findings manually curated by scientists from published literature. Using this mRNA target filter, we conducted a core analysis that identified canonical pathways, diseases and functions, and upstream regulators affected by these mRNA. Canonical pathways and diseases and function annotations were considered statistically significant if the *p*-value was <0.05 using Fisher’s exact test and the absolute value of the activation z-score was two or greater. In these analyses, a positive z-score indicates activation and a negative z-score indicates downregulation. 

## Figures and Tables

**Table 1:** Clinical and Demographics Data.

	Alzheimer Disease (n = 18)	Controls (n = 14)	*p*-Value
Age (years)	73 ± 8	74 ± 9	0.83
Male sex (n, %)	9 (50%)	7 (50%)	0.88
Race/Ethnicity			
White (n, %)	17 (94%)	13 (93%)	n/a
Asian (n, %)	1 (6%)	1 (7%)	n/a
BMI, (kg/m^2^)	24.8 ± 3.5	25.8 ± 4.7	0.52
CDR^®^ Score *	1.25 ± 0.67	0.04 ± 0.13	<0.001
MMSE	18.8 ± 5.4	n/a	n/a
MoCA	n/a	27.6 ± 2.5	n/a
CSF Aβ_1–42_, (pg/mL) *	134.5 ± 38.0	809.6 ± 241.8	<0.001
CSF Total Tau, (pg/mL)	88.4 ± 62.2	75.7 ± 24.0	0.43

CSF Aβ_1–42_ = CSF level of amyloid beta 1–42. CDR^®^ Score = Clinical Dementia Rating. MMSE = Mini-Mental State Exam. MoCA= Montreal Cognitive Assessment. All data are listed as mean ± standard deviation unless otherwise stated. * indicates results were significant with a *p*-value < 0.05.

**Table 2:** Concordant Pathways in Serum and CSF predicted by the Turquoise Module hub miRNAs.

Pathway Name	CSF z-Score	Serum z-Score
CREB Signaling in Neurons	−11.465	−7.832
Synaptogenesis Signaling Pathway	−7.717	−3.578
Gustation Pathway	−6.638	−4.323
GNRH Signaling	−6.38	−4.523
Ephrin Receptor Signaling	−6.14	−2.846
Oxytocin In Brain Signaling Pathway	−6.114	−3.781
SNARE Signaling Pathway	−5.921	−2.795
Synaptic Long-Term Depression	−5.897	−3.111
Cholecystokinin/Gastrin-mediated Signaling	−5.88	−4.768
Regulation of Actin-based Motility by Rho	−5.692	−2.711
NGF Signaling	−5.516	−3.528
Opioid Signaling Pathway	−5.07	−2.828
ERBB Signaling	−5.013	−3
Neurovascular Coupling Signaling Pathway	−4.93	−3.75
GPCR-Mediated Nutrient Sensing in Enteroendocrine Cells	−4.907	−2.496
GDNF Family Ligand-Receptor Interactions	−4.747	−3.578
Neurotrophin/TRK Signaling	−4.382	−4.025
Agrin Interactions at Neuromuscular Junction	−4.315	−2.183
Endocannabinoid Developing Neuron Pathway	−4.258	−2.785
Neuropathic Pain Signaling In Dorsal Horn Neurons	−4.117	−2.357
CNTF Signaling	−3.674	−3.317
Ephrin B Signaling	−3.651	−2.357
ERB2-ERBB3 Signaling	−3.402	−2.183
ERBB4 Signaling	−3.272	−2.673
Oxytocin In Spinal Neurons Signaling Pathway	−3.207	−2.53
Neuroinflammation Signaling Pathway	−3.161	−3.597
CDK5 Signaling	−3.015	−4.017
Dopamine Receptor Signaling	−2.714	−2.236
Dopamine-DARPP32 Feedback in cAMP Signaling	−2.534	−3.333

## Data Availability

The data presented in this study are openly available in NCBI Gene Expression Omnibus (GEO Accession number: GSE242923).

## Supplementary Materials

The following supporting information can be downloaded at: https://www.mdpi.com/article/10.3390/ijms241814024/s1.
