# Supplementary material for: Adipocyte-Derived Small Extracellular Vesicles from Patients with Alzheimer Disease Carry miRNAs Predicted to Target the CREB Signaling Pathway in Neurons
Source: Int J Mol Sci. 2023 Sep 13;24(18):14024. doi: 10.3390/ijms241814024 (PMC10530811; doi:10.3390/ijms241814024)
Supplement: Supplementary file 1 [file ijms-24-14024-s001.zip › Supplemental Figures S1-S3.pdf]

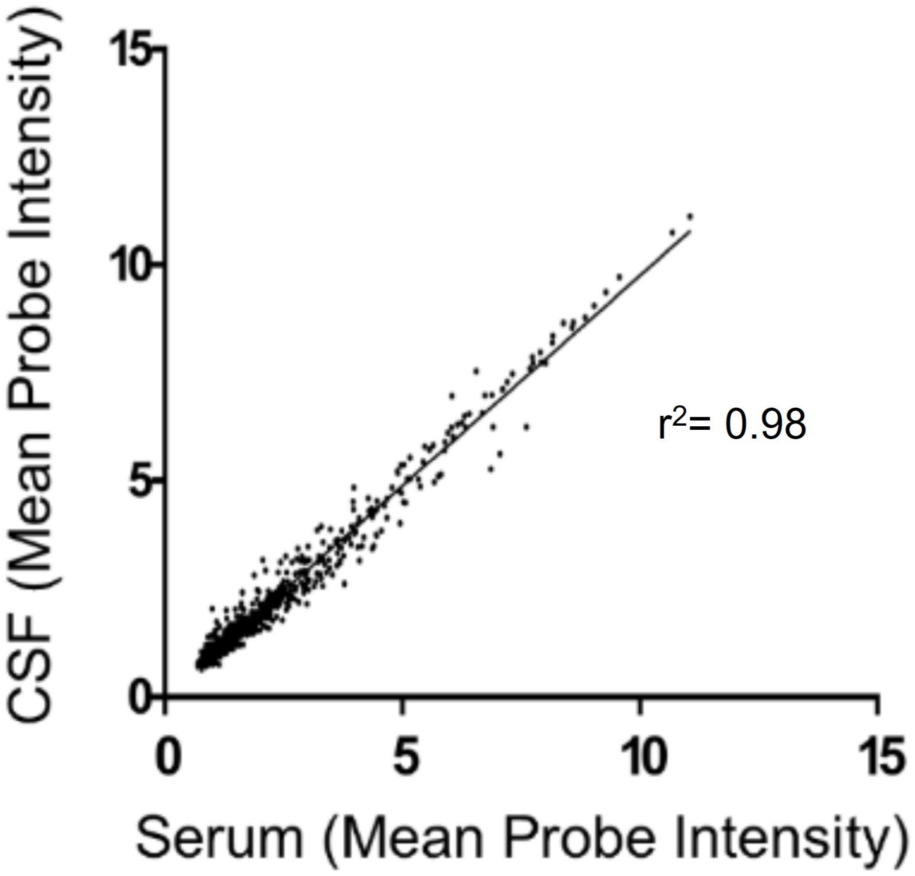

Figure S1: The linear regression of the mean probe intensities for all matched serum and CSF samples.

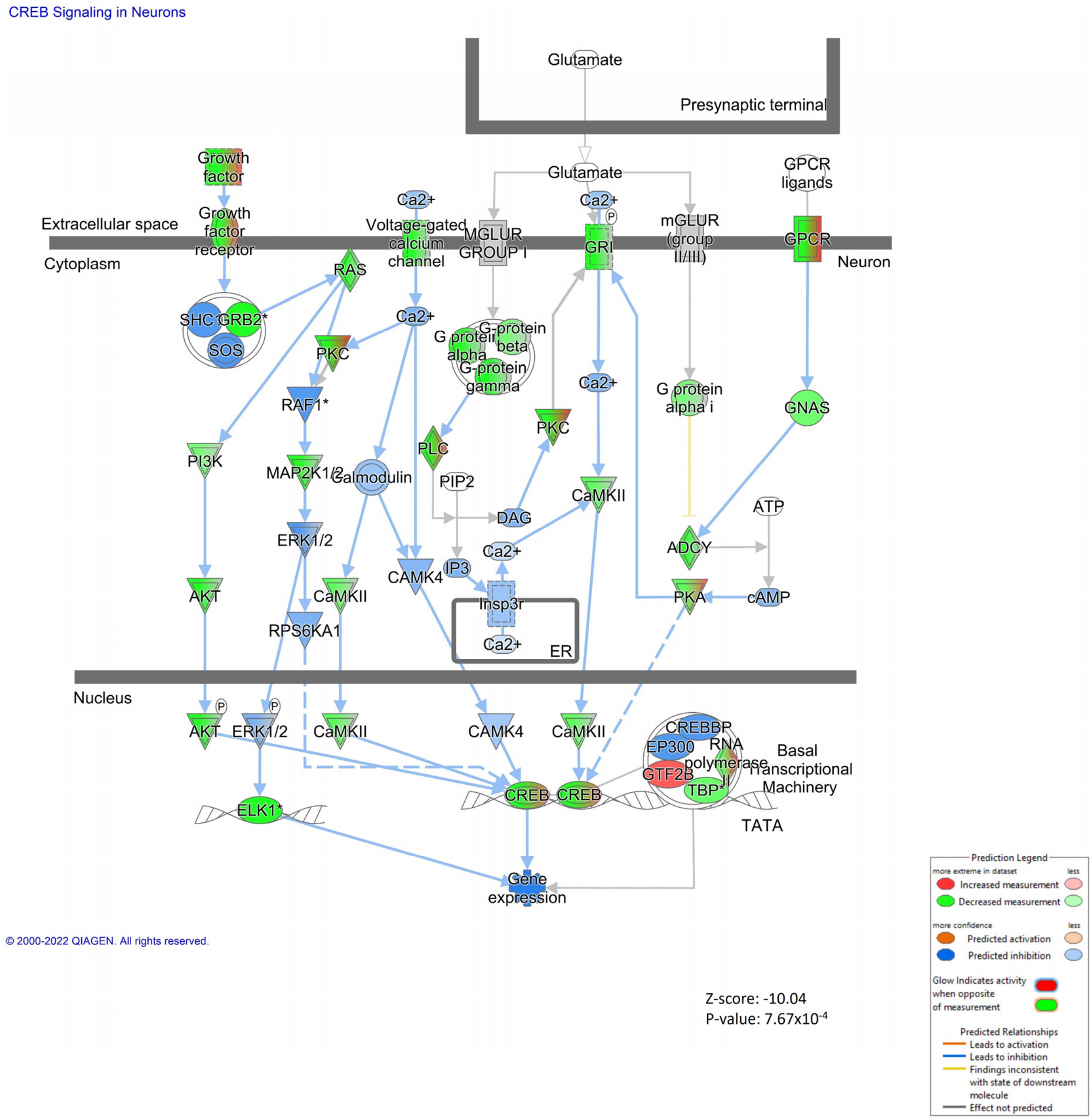

Figure S2. The predicted CREB signaling in neurons pathway based on differentially expressed miRNAs in the serum of AD patients vs. controls.

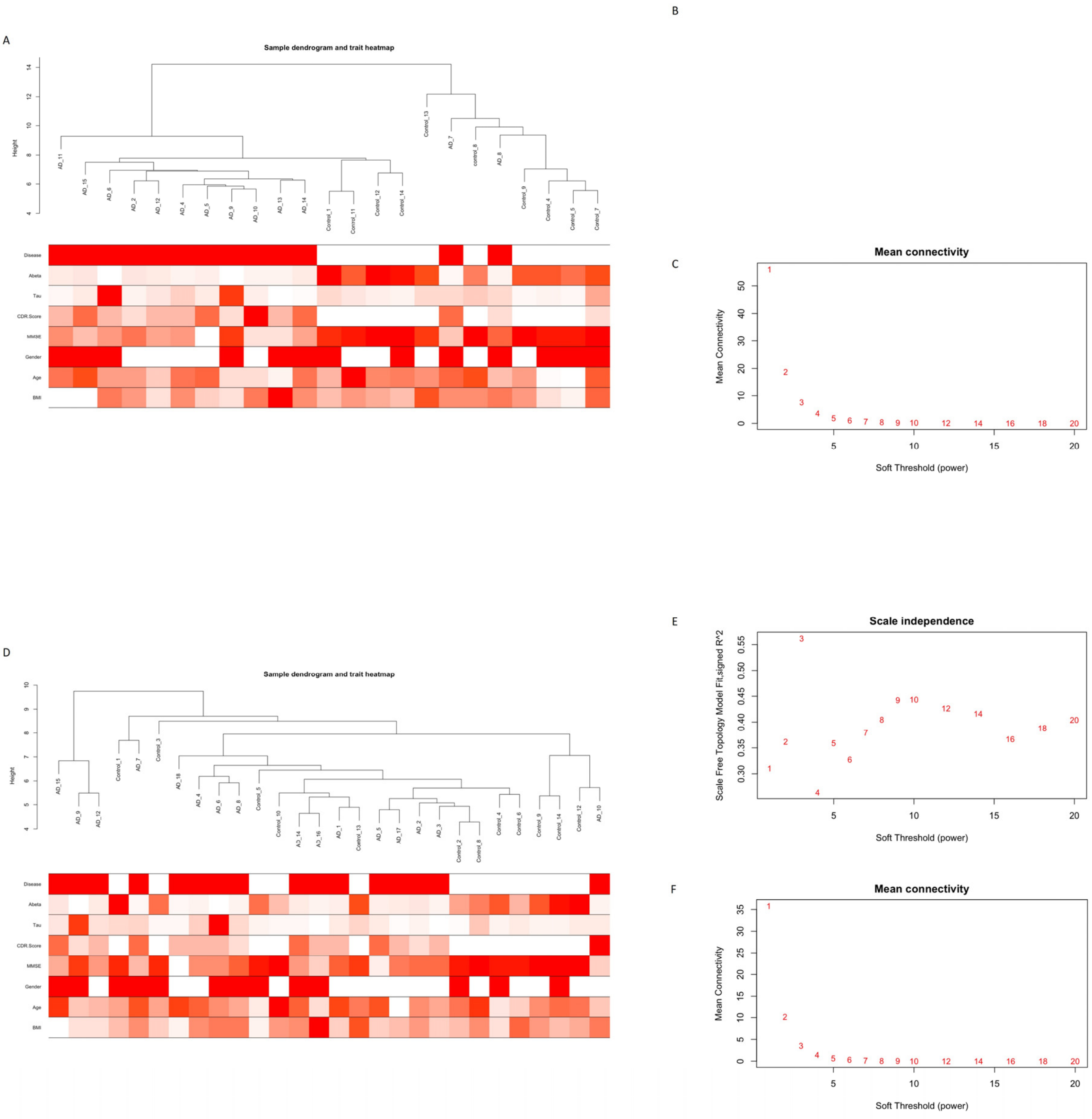

Figure S3. Sample dendrogram and trait heatmap for the (A) CSF and (D) serum. White indicates a low value, and red indicates a high value. (B) The scale-free fit index (y-axis) as a function of the soft-thresholding power (x-axis) for CSF. (C) The mean connectivity (degree, y-axis) as a function of the soft thresholding power in the CSF. (E) The scale-free fit index (y-axis) as a function of the soft-thresholding power (x-axis) for serum. (F) The mean connectivity (degree, y-axis) as a function of the soft thresholding power in the serum.
