# Supplementary material for: Adipocyte-Derived Small Extracellular Vesicles from Patients with Alzheimer Disease Carry miRNAs Predicted to Target the CREB Signaling Pathway in Neurons
Source: Int J Mol Sci. 2023 Sep 13;24(18):14024. doi: 10.3390/ijms241814024 (PMC10530811; doi:10.3390/ijms241814024)
Supplement: Supplementary file 1 [file ijms-24-14024-s001.zip › Supplemental Table S1_CSF.pdf]

Supplemental Table S1. Differentially expressed microRNAs between AD and Control from ad-sEVs isolated from CSF (Fold Change ≥ |1.1|; p- value < 0.1)

Transcript ID	Fold-Change (AD vs. Control)	p-value (AD vs. Control)
hsa-let-7f-5p	1.10149	0.0797757
hsa-miR-1185-5p	-1.12083	0.0897443
hsa-miR-1193	1.15728	0.00823808
hsa-miR-1200	-1.12264	0.0225944
hsa-miR-1202	1.23292	0.072288
hsa-miR-1204	1.13378	0.0249948
hsa-miR-1207-3p	1.23497	0.0117734
hsa-miR-1207-5p	1.42981	0.08967
hsa-miR-1225-3p	1.32327	0.0926715
hsa-miR-1227-3p	1.25447	0.00923388
hsa-miR-1233-5p	1.14433	0.0798651
hsa-miR-1236-5p	1.71123	0.0744683
hsa-miR-1246	1.15991	0.0295699
hsa-miR-1249	1.60308	0.0332896
hsa-miR-1269b	1.20802	0.0118193
hsa-miR-1271-5p	-1.11083	0.0717676
hsa-miR-1276	1.1063	0.0303951
hsa-miR-1277-5p	1.12356	0.0837516
hsa-miR-1278	1.14174	0.0954964
hsa-miR-1284	1.12457	0.0250539
hsa-miR-1286	-1.18097	0.00172318
hsa-miR-1289	1.16315	0.000216639
hsa-miR-1298-5p	1.12638	0.0529002
hsa-miR-1303	-1.11798	0.0715363
hsa-miR-1304-5p	1.14404	0.0938439
hsa-miR-130b-5p	1.10828	0.0908011
hsa-miR-134-5p	-1.15121	0.0534252
hsa-miR-140-3p	1.19881	0.00563168
hsa-miR-15a-3p	1.12117	0.0957291
hsa-miR-183-3p	-1.18998	0.0162964
hsa-miR-188-5p	1.13787	0.0840471
hsa-miR-1911-5p	1.12781	0.0704566
hsa-miR-1915-5p	1.16272	0.0336596
hsa-miR-192-3p	1.14103	0.023333
hsa-miR-193a-5p	-1.12474	0.0353834
hsa-miR-196b-3p	-2.75798	0.0781355

hsa-miR-199a-3p	1.12907	0.0199701
hsa-miR-199b-3p	1.12907	0.0199701
hsa-miR-203b-3p	2.05362	0.0864085
hsa-miR-205-3p	1.17175	0.000930386
hsa-miR-2114-3p	-1.1028	0.0664786
hsa-miR-218-5p	-1.10061	0.0978838
hsa-miR-219a-5p	1.11118	0.0518213
hsa-miR-22-3p	-1.13699	0.032123
hsa-miR-2277-3p	-1.10647	0.0481512
hsa-miR-2278	1.32753	0.07255
hsa-miR-24-2-5p	-1.16259	0.0718464
hsa-miR-25-3p	1.15063	0.0970387
hsa-miR-2681-3p	1.13532	0.00736711
hsa-miR-2682-3p	-1.15513	0.00762352
hsa-miR-2909	-1.13451	0.0141313
hsa-miR-299-5p	1.15752	0.0500452
hsa-miR-302d-5p	-1.10723	0.0405406
hsa-miR-3115	-1.10419	0.0246518
hsa-miR-3117-5p	-1.12834	0.0636762
hsa-miR-3138	-1.11411	0.0868009
hsa-miR-3150b-5p	1.18217	0.0743547
hsa-miR-3151-3p	1.19086	0.0240769
hsa-miR-3154	1.12643	0.0767281
hsa-miR-3156-5p	1.16621	0.0211226
hsa-miR-3160-5p	-1.13809	0.0390836
hsa-miR-3161	-1.14894	0.0445998
hsa-miR-3164	1.49017	0.0806687
hsa-miR-3180-5p	1.21579	0.0872178
hsa-miR-3200-3p	-1.12268	0.0187395
hsa-miR-3200-5p	-1.11283	0.0744283
hsa-miR-3201	1.3472	0.0931715
hsa-miR-323a-3p	1.12239	0.0772629
hsa-miR-337-3p	-1.27636	0.0286771
hsa-miR-3610	-1.13102	0.0531739
hsa-miR-3616-3p	1.13125	0.0858092
hsa-miR-3652	1.16674	0.0751219
hsa-miR-3664-3p	-1.18027	0.0827643
hsa-miR-3666	-1.39092	0.0109364
hsa-miR-3677-5p	-1.12014	0.0637666
hsa-miR-3679-5p	2.88778	0.00284932
hsa-miR-3684	-1.11747	0.0171116

hsa-miR-3689d	1.10963	0.0535744
hsa-miR-373-5p	-1.17872	0.0372202
hsa-miR-374b-3p	1.1168	0.0663256
hsa-miR-376a-3p	1.84066	0.00411828
hsa-miR-378a-3p	-1.13621	0.0293736
hsa-miR-380-5p	-1.13177	0.0316654
hsa-miR-3911	-1.22416	0.0330252
hsa-miR-3915	1.24666	0.0626638
hsa-miR-3918	1.13819	0.0796406
hsa-miR-3925-3p	1.10264	0.0158238
hsa-miR-3945	-1.13028	0.0887894
hsa-miR-3977	-1.17009	0.00448556
hsa-miR-409-3p	1.2816	0.0340593
hsa-miR-425-5p	1.12258	0.0526108
hsa-miR-4263	-1.16337	0.0376118
hsa-miR-4274	1.51581	0.00799523
hsa-miR-4275	1.12375	0.0592624
hsa-miR-4281	1.51877	0.0464617
hsa-miR-4297	1.11897	0.0822888
hsa-miR-4310	1.60319	0.0364734
hsa-miR-4314	1.67514	0.0902983
hsa-miR-433-5p	-1.12193	0.0263853
hsa-miR-4426	-1.16037	0.00381748
hsa-miR-4432	-1.56716	0.082786
hsa-miR-4439	1.11183	0.0480312
hsa-miR-4482-3p	-1.11871	0.0698481
hsa-miR-4488	-1.14924	0.0436392
hsa-miR-4490	-1.24662	0.00348656
hsa-miR-4492	1.13125	0.0773364
hsa-miR-4497	-1.1841	0.0691412
hsa-miR-4503	1.18233	0.0420616
hsa-miR-4504	1.11327	0.0455249
hsa-miR-4505	1.22576	0.0285877
hsa-miR-4514	1.46082	0.0941025
hsa-miR-4524a-3p	1.18912	0.0530895
hsa-miR-4532	-1.16488	0.0666016
hsa-miR-4539	-1.14262	0.0772943
hsa-miR-4634	-1.19727	0.00183033
hsa-miR-4640-5p	1.21051	0.0276935
hsa-miR-4642	1.34714	0.0506389
hsa-miR-4646-3p	1.14489	0.095003

hsa-miR-4652-3p	1.20316	0.053275
hsa-miR-4659a-3p	1.10176	0.0066628
hsa-miR-4661-3p	-1.12973	0.0544202
hsa-miR-4662a-3p	-1.15278	0.0961803
hsa-miR-4666a-5p	-1.29652	0.0521443
hsa-miR-4670-5p	-1.11424	0.048036
hsa-miR-4685-5p	1.12838	0.081669
hsa-miR-4705	1.13906	0.012456
hsa-miR-4707-3p	1.35241	0.0101257
hsa-miR-4712-5p	1.11696	0.0570727
hsa-miR-4714-3p	-1.19155	0.0259763
hsa-miR-4716-3p	1.82986	0.0609547
hsa-miR-4721	-1.13772	0.0488907
hsa-miR-4725-3p	1.18328	0.0333919
hsa-miR-4735-3p	1.17601	0.0234188
hsa-miR-4739	1.22884	0.0151969
hsa-miR-4740-5p	1.10451	0.027732
hsa-miR-4745-3p	1.13297	0.00877104
hsa-miR-4747-5p	-1.12899	0.0671991
hsa-miR-4750-3p	1.43774	0.0742748
hsa-miR-4750-5p	1.17524	0.0557961
hsa-miR-4769-3p	1.46911	0.0282526
hsa-miR-4769-5p	1.15076	0.0825036
hsa-miR-4774-3p	1.1526	0.096135
hsa-miR-4779	1.82312	0.0435946
hsa-miR-4792	1.15468	0.0345627
hsa-miR-4799-3p	-1.10651	0.0897789
hsa-miR-483-5p	2.20327	0.000788546
hsa-miR-496	-1.1327	0.0215909
hsa-miR-497-3p	1.12347	0.0341588
hsa-miR-497-5p	1.11031	0.074853
hsa-miR-499a-3p	1.12465	0.0717019
hsa-miR-5002-5p	1.11683	0.0246336
hsa-miR-500b-5p	-1.14168	0.0736747
hsa-miR-501-3p	-1.17764	0.0292446
hsa-miR-5010-5p	1.28731	0.0564004
hsa-miR-503-5p	1.17773	0.0327721
hsa-miR-509-5p	-1.12082	0.0688064
hsa-miR-514a-5p	1.12093	0.0351929
hsa-miR-517-5p	-1.1584	0.0372689
hsa-miR-518a-5p	-1.12217	0.0757206

hsa-miR-5195-5p	1.23232	0.0581709
hsa-miR-520f-5p	1.12902	0.0107023
hsa-miR-524-5p	1.36801	0.0399535
hsa-miR-526b-5p	1.22401	0.0350348
hsa-miR-527	-1.12217	0.0757206
hsa-miR-548ab	1.10737	0.0300979
hsa-miR-548ah-5p	-1.15061	0.00639149
hsa-miR-548w	1.18628	0.0041055
hsa-miR-5580-3p	-1.13095	0.0801792
hsa-miR-5583-3p	1.11909	0.0506909
hsa-miR-5585-3p	1.14297	0.0729767
hsa-miR-5587-3p	1.10895	0.0809632
hsa-miR-5589-5p	1.1418	0.0813504
hsa-miR-5591-5p	1.11351	0.084305
hsa-miR-5700	-1.13389	0.0461513
hsa-miR-576-3p	1.91035	0.00682479
hsa-miR-604	-1.27363	0.0397478
hsa-miR-605-3p	-1.13923	0.0737419
hsa-miR-6083	-1.12105	0.0815203
hsa-miR-613	-1.11805	0.0346498
hsa-miR-6131	1.19858	0.0240259
hsa-miR-625-3p	1.51464	0.0145551
hsa-miR-625-5p	1.89289	0.0761664
hsa-miR-627-3p	-15.4881	0.0450433
hsa-miR-630	1.14003	0.0143177
hsa-miR-636	3.26459	0.0578967
hsa-miR-637	-1.46533	0.0167417
hsa-miR-642a-3p	1.86326	0.00148693
hsa-miR-6500-5p	1.1519	0.0254942
hsa-miR-6501-5p	-1.1716	0.0378687
hsa-miR-6506-5p	1.19239	0.0226866
hsa-miR-6513-5p	1.12792	0.0657771
hsa-miR-658	1.20795	0.0604973
hsa-miR-659-3p	1.28872	0.0177836
hsa-miR-664a-5p	1.90853	0.0222939
hsa-miR-668-3p	1.26753	0.0464705
hsa-miR-6722-3p	-1.20034	0.0322632
hsa-miR-6732-5p	1.48018	0.0437548
hsa-miR-6741-5p	-1.16261	0.0840121
hsa-miR-6747-3p	-1.81023	0.0921802
hsa-miR-6747-5p	1.2461	0.0119783

hsa-miR-675-3p	-1.21038	0.0346676
hsa-miR-675-5p	-1.14107	0.0456228
hsa-miR-6753-3p	-1.14818	0.0284435
hsa-miR-6757-3p	1.16511	0.037885
hsa-miR-6760-3p	1.61699	0.0462611
hsa-miR-6768-5p	1.35191	0.0273011
hsa-miR-6775-5p	1.81621	0.0140146
hsa-miR-6776-5p	1.16279	0.0972545
hsa-miR-6783-5p	1.68876	0.0550799
hsa-miR-6788-5p	1.13592	0.0918777
hsa-miR-6796-3p	1.3544	0.0594585
hsa-miR-6797-3p	1.34465	0.0386018
hsa-miR-6797-5p	1.65365	0.014762
hsa-miR-6798-3p	1.83546	0.011291
hsa-miR-6805-3p	1.42038	0.0135338
hsa-miR-6807-3p	1.12518	0.0830775
hsa-miR-6814-5p	-1.16906	0.00564461
hsa-miR-6815-3p	1.11802	0.0178991
hsa-miR-6818-5p	1.14035	0.0631906
hsa-miR-6829-5p	-1.17	0.0779249
hsa-miR-6834-5p	-1.11906	0.0686274
hsa-miR-6836-3p	1.44506	0.0138726
hsa-miR-6840-5p	1.20196	0.0956448
hsa-miR-6847-3p	1.17069	0.071335
hsa-miR-6854-3p	1.14838	0.0141964
hsa-miR-6855-5p	1.19263	0.0328708
hsa-miR-6856-3p	1.10527	0.0575459
hsa-miR-6858-3p	1.34247	0.0620538
hsa-miR-6861-5p	1.13272	0.0793974
hsa-miR-6863	1.12129	0.0215275
hsa-miR-6870-5p	1.28963	0.0288787
hsa-miR-6874-3p	-1.30291	0.0414019
hsa-miR-6886-3p	-1.3017	0.0292102
hsa-miR-6888-5p	-1.11059	0.0865319
hsa-miR-7111-5p	-1.17043	0.0649289
hsa-miR-7150	1.19642	0.0841284
hsa-miR-7152-3p	-1.11317	0.0416619
hsa-miR-7153-3p	-1.10096	0.0484984
hsa-miR-7156-3p	1.37145	0.000988049
hsa-miR-7157-5p	1.11079	0.0765129
hsa-miR-7158-3p	1.12214	0.0508187

hsa-miR-7160-3p	1.1772	0.0666053
hsa-miR-7162-3p	1.15097	0.0139879
hsa-miR-7515	1.32434	0.0538987
hsa-miR-766-3p	1.21472	0.0164327
hsa-miR-766-5p	-1.29776	0.0177525
hsa-miR-7855-5p	1.22003	0.0329069
hsa-miR-8059	1.32572	0.0662445
hsa-miR-8087	1.13113	0.0571774
hsa-miR-924	-1.23754	0.0569147
hsa-miR-95-5p	-1.10425	0.0209264
