# Supplementary material for: Adipocyte-Derived Small Extracellular Vesicles from Patients with Alzheimer Disease Carry miRNAs Predicted to Target the CREB Signaling Pathway in Neurons
Source: Int J Mol Sci. 2023 Sep 13;24(18):14024. doi: 10.3390/ijms241814024 (PMC10530811; doi:10.3390/ijms241814024)
Supplement: Supplementary file 1 [file ijms-24-14024-s001.zip › Supplemental Table S2_serum.pdf]

**Supplemental Table S2.** Differentially expressed microRNAs between AD and Control from ad-sEVs isolated from serum (Fold Change ≥ |1.1|; p- value < 0.1)

| Transcript ID | Fold-Change(AD vs. Control) | p-Value (AD vs. Control) |
|---|---|---|
| hsa-let-7a-5p | -1.13177 | 0.0277742 |
| hsa-let-7c-3p | 1.13721 | 0.0812868 |
| hsa-miR-106b-5p | -1.1333 | 0.0859059 |
| hsa-miR-10b-3p | 1.2967 | 0.0826638 |
| hsa-miR-1199-3p | 1.22177 | 0.0043076 |
| hsa-miR-1199-5p | 1.10667 | 0.0293895 |
| hsa-miR-1236-3p | 1.24025 | 0.0347887 |
| hsa-miR-1237-3p | 1.13805 | 0.0815098 |
| hsa-miR-1250-3p | 1.10404 | 0.0314444 |
| hsa-miR-1252-3p | 1.11744 | 0.022432 |
| hsa-miR-1255b-5p | 1.44971 | 0.0475576 |
| hsa-miR-125b-2-3p | 1.10283 | 0.0428087 |
| hsa-miR-1286 | 1.12377 | 0.0317488 |
| hsa-miR-1303 | 1.11739 | 0.0650393 |
| hsa-miR-1343-3p | 1.88688 | 0.0266127 |
| hsa-miR-138-1-3p | -1.70133 | 0.095376 |
| hsa-miR-138-5p | 1.12729 | 0.0573332 |
| hsa-miR-139-5p | 1.15103 | 0.0338362 |
| hsa-miR-185-5p | 1.10646 | 0.0326375 |
| hsa-miR-186-3p | -1.1369 | 0.0628287 |
| hsa-miR-187-5p | -1.24044 | 0.0205276 |
| hsa-miR-1908-5p | 1.17038 | 0.0963102 |
| hsa-miR-190a-5p | -1.11827 | 0.087159 |
| hsa-miR-1913 | 1.24058 | 0.0488713 |
| hsa-miR-1914-5p | 1.41906 | 0.0894655 |
| hsa-miR-196a-5p | -1.18471 | 0.0560181 |
| hsa-miR-197-5p | 1.19797 | 0.0338943 |
| hsa-miR-200c-5p | -1.10768 | 0.0368413 |
| hsa-miR-208a-5p | -1.13035 | 0.0977729 |
| hsa-miR-21-5p | -1.15849 | 0.000795994 |
| hsa-miR-217 | 2.49834 | 0.0483326 |
| hsa-miR-224-3p | 1.10096 | 0.0694768 |
| hsa-miR-224-5p | 1.10065 | 0.07306 |
| hsa-miR-2276-5p | 1.10549 | 0.0794444 |
| hsa-miR-24-2-5p | -1.29857 | 0.0256113 |
| hsa-miR-26a-5p | 1.13122 | 0.0498957 |

| | | |
|---|---|---|
| hsa-miR-297 | -1.16488 | 0.00473434 |
| hsa-miR-301a-3p | 1.11862 | 0.0375706 |
| hsa-miR-301b | -1.10113 | 0.0573039 |
| hsa-miR-302f | -1.11358 | 0.0651033 |
| hsa-miR-3064-3p | -1.24351 | 0.0411377 |
| hsa-miR-31-3p | 1.12535 | 0.0497808 |
| hsa-miR-3117-5p | 1.11547 | 0.0378873 |
| hsa-miR-3158-5p | 1.11649 | 0.0340265 |
| hsa-miR-3160-3p | 1.33344 | 0.00741704 |
| hsa-miR-3166 | 1.14363 | 0.012546 |
| hsa-miR-3175 | 1.22684 | 0.0039855 |
| hsa-miR-3177-5p | 1.13193 | 0.0279692 |
| hsa-miR-3188 | -1.1435 | 0.0302046 |
| hsa-miR-323a-5p | 1.1365 | 0.0618984 |
| hsa-miR-33a-3p | -1.11106 | 0.0898195 |
| hsa-miR-3605-3p | -1.11726 | 0.0731036 |
| hsa-miR-3605-5p | 1.18348 | 0.0744513 |
| hsa-miR-3607-5p | -1.11851 | 0.0200367 |
| hsa-miR-3612 | -1.10526 | 0.00594083 |
| hsa-miR-3620-5p | -1.22408 | 0.0941493 |
| hsa-miR-3622b-3p | -1.10656 | 0.00389375 |
| hsa-miR-3655 | -1.27514 | 0.00768299 |
| hsa-miR-3678-5p | 1.37184 | 0.0146815 |
| hsa-miR-3679-3p | 1.62929 | 0.00538746 |
| hsa-miR-3692-3p | 1.15509 | 0.0266596 |
| hsa-miR-374b-3p | -1.10538 | 0.06478 |
| hsa-miR-376a-2-5p | -1.11308 | 0.024737 |
| hsa-miR-3915 | 1.26589 | 0.0621354 |
| hsa-miR-3921 | 1.52714 | 0.0717592 |
| hsa-miR-3926 | 1.29811 | 0.0700366 |
| hsa-miR-3943 | 1.32304 | 0.0139314 |
| hsa-miR-3944-3p | 1.11612 | 0.0938358 |
| hsa-miR-3944-5p | 1.15203 | 0.0283838 |
| hsa-miR-409-5p | -1.2437 | 0.00159462 |
| hsa-miR-4259 | -1.20347 | 0.00386513 |
| hsa-miR-4273 | -1.19161 | 0.00845012 |
| hsa-miR-4279 | 1.11875 | 0.029903 |
| hsa-miR-429 | -1.17203 | 0.0952935 |
| hsa-miR-4294 | 1.16799 | 0.00220369 |
| hsa-miR-4306 | -1.13151 | 0.0483574 |

| | | |
|---|---|---|
| hsa-miR-4329 | 1.1205 | 0.0783501 |
| hsa-miR-4419a | -1.19907 | 0.00477339 |
| hsa-miR-4421 | -1.14277 | 0.0657246 |
| hsa-miR-4422 | 2.53444 | 0.0477607 |
| hsa-miR-4424 | 1.2313 | 0.00102055 |
| hsa-miR-4446-5p | 1.20764 | 0.00599809 |
| hsa-miR-4450 | 1.11036 | 0.0911047 |
| hsa-miR-4459 | 1.13035 | 0.0963205 |
| hsa-miR-4464 | -1.10148 | 0.0776449 |
| hsa-miR-4471 | 1.10779 | 0.0877703 |
| hsa-miR-4482-5p | 1.12715 | 0.0707577 |
| hsa-miR-4505 | -1.17037 | 0.0282409 |
| hsa-miR-451b | 1.10779 | 0.0787897 |
| hsa-miR-4524b-5p | -1.19024 | 0.000287977 |
| hsa-miR-4528 | -1.13825 | 0.0155555 |
| hsa-miR-4529-3p | 1.21225 | 0.020685 |
| hsa-miR-4530 | -1.1546 | 0.0383548 |
| hsa-miR-4538 | -1.12579 | 0.0275112 |
| hsa-miR-455-3p | -1.5558 | 0.0254584 |
| hsa-miR-4643 | 1.15976 | 0.0389565 |
| hsa-miR-4666a-5p | 1.20238 | 0.0574589 |
| hsa-miR-4670-5p | 1.13946 | 0.0448325 |
| hsa-miR-4705 | 1.1031 | 0.0571685 |
| hsa-miR-4716-5p | 1.35962 | 0.0528678 |
| hsa-miR-4723-3p | 1.30219 | 0.0916889 |
| hsa-miR-4725-3p | 1.1259 | 0.0980072 |
| hsa-miR-4731-5p | 1.10242 | 0.01535 |
| hsa-miR-4733-3p | -1.12511 | 0.0948208 |
| hsa-miR-4734 | -1.14392 | 0.0269979 |
| hsa-miR-4735-3p | 1.13758 | 0.0205188 |
| hsa-miR-4742-5p | -1.20726 | 0.0713683 |
| hsa-miR-4743-3p | 1.12787 | 0.032405 |
| hsa-miR-4747-3p | 1.13943 | 0.0190976 |
| hsa-miR-4749-3p | 1.44526 | 0.0798796 |
| hsa-miR-4753-5p | 1.29308 | 0.0215797 |
| hsa-miR-4756-5p | 2.01263 | 0.0856924 |
| hsa-miR-4763-5p | 1.13363 | 0.0511754 |
| hsa-miR-4767 | 1.14421 | 0.00608644 |
| hsa-miR-4768-3p | -1.13528 | 0.0220058 |
| hsa-miR-4773 | 1.17105 | 0.0354102 |
| hsa-miR-487a-5p | -1.13877 | 0.0253692 |

| | | |
|---|---|---|
| hsa-miR-488-3p | -1.14613 | 0.0324509 |
| hsa-miR-497-5p | 1.14022 | 0.0667661 |
| hsa-miR-5004-3p | 1.10906 | 0.0290855 |
| hsa-miR-5008-3p | 1.18026 | 0.0312064 |
| hsa-miR-5009-5p | 1.13581 | 0.0830887 |
| hsa-miR-5047 | 1.10249 | 0.0891322 |
| hsa-miR-505-3p | -1.11684 | 0.0835975 |
| hsa-miR-5096 | 1.10076 | 0.0358596 |
| hsa-miR-512-3p | -1.10807 | 0.0749698 |
| hsa-miR-516b-5p | 1.14368 | 0.0186076 |
| hsa-miR-5187-3p | 1.10482 | 0.0811398 |
| hsa-miR-5197-5p | -1.20511 | 0.0827939 |
| hsa-miR-519d-5p | -1.13717 | 0.0862293 |
| hsa-miR-545-3p | 1.12151 | 0.0350689 |
| hsa-miR-548av-3p | 1.26309 | 0.00348708 |
| hsa-miR-548k | -1.16243 | 0.0050528 |
| hsa-miR-548v | -1.15262 | 0.00815877 |
| hsa-miR-5572 | -1.25752 | 0.00260632 |
| hsa-miR-5581-3p | 1.16398 | 0.0275883 |
| hsa-miR-5581-5p | -1.11488 | 0.0730323 |
| hsa-miR-564 | 1.11734 | 0.0192397 |
| hsa-miR-5707 | -1.12952 | 0.0144714 |
| hsa-miR-579-3p | -1.12952 | 0.087975 |
| hsa-miR-591 | 1.10255 | 0.0318109 |
| hsa-miR-601 | 1.16821 | 0.0254619 |
| hsa-miR-603 | -1.27833 | 0.0440589 |
| hsa-miR-6069 | 1.54165 | 0.000552017 |
| hsa-miR-6085 | 1.37303 | 0.0422034 |
| hsa-miR-6087 | 1.58062 | 0.079137 |
| hsa-miR-6089 | 1.96894 | 0.0952743 |
| hsa-miR-6129 | -1.12131 | 0.0908769 |
| hsa-miR-621 | 1.13556 | 0.036581 |
| hsa-miR-622 | -1.12012 | 0.0756841 |
| hsa-miR-624-3p | 1.10669 | 0.00659693 |
| hsa-miR-654-5p | 1.14644 | 0.0708922 |
| hsa-miR-660-5p | -1.10486 | 0.0364328 |
| hsa-miR-670-5p | -1.12571 | 0.0256287 |
| hsa-miR-6716-3p | 1.42473 | 0.00359851 |
| hsa-miR-6760-3p | 1.47814 | 0.0243843 |
| hsa-miR-6761-5p | -1.10375 | 0.0767089 |
| hsa-miR-6770-5p | 1.12869 | 0.0736925 |

| | | |
|---|---|---|
| hsa-miR-6786-3p | 1.29276 | 0.0110051 |
| hsa-miR-6787-3p | -1.11356 | 0.0775895 |
| hsa-miR-6793-3p | 1.23888 | 0.0820825 |
| hsa-miR-6793-5p | 1.14179 | 0.014394 |
| hsa-miR-6798-3p | 1.41431 | 0.0475633 |
| hsa-miR-6812-3p | 1.3314 | 0.0130667 |
| hsa-miR-6817-3p | -1.15096 | 0.0727983 |
| hsa-miR-6819-5p | 1.18943 | 0.0600201 |
| hsa-miR-6823-5p | 1.10534 | 0.0809655 |
| hsa-miR-6824-3p | 1.17036 | 0.0384258 |
| hsa-miR-6825-5p | 1.14079 | 0.0724082 |
| hsa-miR-6829-3p | -1.17579 | 0.0234138 |
| hsa-miR-6837-3p | 1.35107 | 0.0131251 |
| hsa-miR-6838-5p | 1.13749 | 0.0909215 |
| hsa-miR-6842-5p | 1.14788 | 0.00748683 |
| hsa-miR-6846-3p | 1.16616 | 0.0679891 |
| hsa-miR-6846-5p | -1.15192 | 0.0477337 |
| hsa-miR-6865-3p | -1.1136 | 0.0657678 |
| hsa-miR-6868-5p | 1.16826 | 0.075809 |
| hsa-miR-6869-3p | 1.1009 | 0.0239604 |
| hsa-miR-6875-3p | 1.13177 | 0.0325637 |
| hsa-miR-6890-3p | 1.22677 | 0.0542669 |
| hsa-miR-7113-3p | 1.99159 | 0.00621289 |
| hsa-miR-767-3p | 1.3135 | 0.0120529 |
| hsa-miR-7706 | 1.11321 | 0.047162 |
| hsa-miR-8083 | -1.11311 | 0.0170642 |
| hsa-miR-874-5p | 1.18564 | 0.0716315 |
| hsa-miR-888-5p | 1.7567 | 0.0167286 |
| hsa-miR-891a-3p | -1.12629 | 0.0541082 |
| hsa-miR-93-5p | 1.33629 | 0.0262484 |
| hsa-miR-95-5p | 1.16344 | 0.0267385 |
